# An Easy Method for Pressure Measurement in Microchannels Using Trapped Air Compression in a One-End-Sealed Capillary

**DOI:** 10.3390/mi11100914

**Published:** 2020-09-30

**Authors:** Feng Shen, Mingzhu Ai, Jianfeng Ma, Zonghe Li, Sen Xue

**Affiliations:** 1 Faculty of Materials and Manufacturing, Beijing University of Technology, Beijing 100124, China; aimingzhu3586@163.com (M.A.); lizonghe@emails.bjut.edu.cn (Z.L.); 2 Beijing Key Laboratory of Advanced Manufacturing Technology, Beijing University of Technology, Beijing 100124, China; 3 Department of Engineering Mechanics, Tsinghua University, Beijing 100084, China; xues19@mails.tsinghua.edu.cn

**Keywords:** microfluidics, pressure measurement, capillary, trapped air, air-liquid interface

## Abstract

Pressure is one basic parameter involved in microfluidic systems. In this study, we developed an easy capillary-based method for measuring fluid pressure at one or multiple locations in a microchannel. The principal component is a commonly used capillary (inner diameter of 400 μm and 95 mm in length), with one end sealed and calibrated scales on it. By reading the height (*h*) of an air-liquid interface, the pressure can be measured directly from a table, which is calculated using the ideal gas law. Many factors that affect the relationship between the trapped air volume and applied pressure (*p_applied_*) have been investigated in detail, including the surface tension, liquid gravity, air solubility in water, temperature variation, and capillary diameters. Based on the evaluation of the experimental and simulation results of the pressure, combined with theoretical analysis, a resolution of about 1 kPa within a full-scale range of 101.6–178 kPa was obtained. A pressure drop (Δ*p*) as low as 0.25 kPa was obtained in an operating range from 0.5 kPa to 12 kPa. Compared with other novel, microstructure-based methods, this method does not require microfabrication and additional equipment. Finally, we use this method to reasonably analyze the nonlinearity of the flow-pressure drop relationship caused by channel deformation. In the future, this one-end-sealed capillary could be used for pressure measurement as easily as a clinical thermometer in various microfluidic applications.

## 1. Introduction

With the rapid development of microfluidics or lab-on-a-chip systems [1,2,3,4], pressure measurement in microchannels is becoming more and more crucial for fundamental understanding and precise control of fluid flows on microscales and for further development of microfluidic devices [5,6,7,8,9,10,11]. First, pressure as one of the most important parameters could directly determine the flow behaviors of cells, particles, droplets, and chemical reagents in microchannels. For example, precisely regulated pressure can realize cell culture, particle focusing and separation, and droplet generation and transport [12,13,14,15,16]. Second, pressure can also be used to characterize the mechanical properties in microfluidic systems, such as bacterial biofilms deflection and cell transformation [17,18,19,20,21,22]. Third, pressure sensors can also be used for implantable pressure monitoring, such as blood, intraocular, and intracranial pressures [23,24]. However, due to the limited range of microchannel dimensions, it is very difficult to integrate an additional pressure-sensing functionality without disturbing the flow field [25,26,27]. Therefore, measuring pressure inside microchannels is challenging and novel methods are demanded [28,29,30,31].

Several methods have been proposed in the literature for pressure measurement inside microchannels [25,26,27,28,29,30,31,32,33,34,35,36,37,38,39,40,41,42,43,44,45]. The majority of these published approaches are mechanical in nature and primarily use a membrane that deflects as a function of the applied pressure [32,33,34,35,36,37,38,39,40,41,42,43,44,45]. The membrane deformation and applied pressure can be detected through a system of capacitive [32,33,34], piezo-resistive [35,36,37], or optical sensors [38,39,40,41,42,43,44,45]. While these mechanical methods show high sensitivity and precision, they typically require a complicated microfabrication process on microfluidic devices and additional signal processing for operation [46]. For example, electronic components are required for electrical measurement methods, making integration and packaging a challenging and expensive task, while optical methods usually require a sophisticated readout setup (e.g., interferometry and confocal fluorescence microscopy) to obtain images and complicated analysis steps to estimate the pressure [27,28,29]. Therefore, seamless chip integration for these methods is challenging. 

In order to simplify the structure, membraneless pressure methods have also been proposed [30,47,48,49,50,51,52,53,54,55,56,57]. According to the measuring position, these methods can be generally classified into two categories. The first category is direct measurement inside the microchannel, for example, the pressure-sensitive paint (PSP) method using luminescence emitted from air pressure-sensitive molecules [30,47,48,49,50] and the deformable elastic microballoon method [51], both of which are optical. The second category is based on a sealed side channel connected to the microchannel at the location where the pressure is to be measured, for example, the flexible liquid metal electrodes-based capacitive method [52] and trapped air compression methods [30,53,54,55,56,57]. Srivastava and Burns [54] developed an easy method for measuring the pressure of liquid and air by monitoring the movement of a liquid–air interface as it compresses air trapped inside a one-end-sealed side channel connected with a chamber. The pressure of the trapped air can be calculated by applying the ideal gas law, where the air volume changes can be calculated according to the air-liquid interface. By using two sealed side channels, multiplex pressure measurements at different positions can be achieved. Since this method primarily uses a sealed side channel, it does not require additional fabrication steps and can be readily integrated [54]. Moreover, this method does not require large-scale instruments to obtain images and complicated analysis steps to estimate the pressure. However, this method is not suitable for permeable substrates such as polydimethylsiloxane (PDMS) substrates because evaporation of the trapped liquid plug may lead to reading errors. Using the trapped air method, Kim et al. [55] reported a rapid-prototyped on-chip vacuum gauge for measuring subatmospheric pressure utilizing volumetric expansion of air trapped in a sealed chamber. Similarly, Suter et al. [53] microfabricated two parallel titanium electrodes along the side channel to monitor the capacitance changes between them induced by the displacement of the air-liquid interface (meniscus). They also investigated the influences of meniscus forces, capacitive sensitivity and gas exchange rate on the measurement accurate over a long time. Hoera et al. [30] developed an all-glass pressure sensor by depositing a PSP film on the interior surface of a sealed side channel with trapped air. By assessing the pressure dependency of the luminescence, this optically readable sensor enables reliable measurement of gas pressure and flow rate-induced liquid backpressure at various positions along a microchannel.

Despite the advancements, pressure measurements on microscales are still going to be challenging and open-ended topics in the near future [56,57,58,59,60]. For practical purposes, a universal pressure sensor should be straightforward and repeatable, which means that the sensor can be integrated in one existing microfluidic chip with minimal alterations and can be reused for other chips without recalibration and more microfabrication. These pressure-measuring devices usually cannot be integrated on an actual chip channel due to various reasons: complicated calibration processes, recalibration for different microfluidic chips, and lack of practicality and commercialization.

In this study, we demonstrate an easy one-end-sealed capillary pressure sensor using trapped air compression, which is inspired by the previous studies of References [53,54,55]. Instead of using the microfabricated side channel and chamber, we just use a common glass capillary (diameter of 400 μm and 95 mm in length) with one-end sealed and scales on it, which is impermeable and can be fabricated very easily in every laboratory. Moreover, as the position of the air-liquid interface (*h*) can be read by eyes directly and no additional equipment (e.g., microscope) is needed, our pressure measurement method is easy and can be used conveniently. The effects of various factors on the accuracy of our method have been investigated and the relationship between the pressure and air-liquid interface have been calculated in detail.

## 2. Materials and Methods 

**Figure 1:**
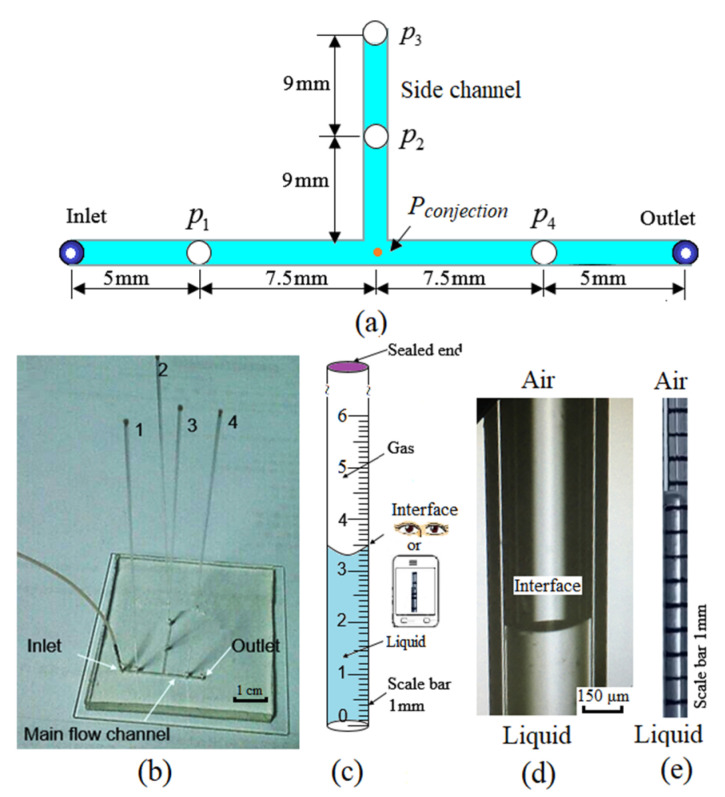
An easy capillary-based pressure measurement method: (**a**) schematic of the T-shaped microchannel with four holes, where the vertical channel is used as a sealed side channel; (**b**) snapshot of the microfluidic chip with four sensing capillaries; (**c**) schematic of the one-end-sealed capillary with scales which can be read by eyes or a smart phone; (**d**) snapshot of the liquid–air interface in the glass capillary recorded by using a high-speed microscopic imaging system (Keyence, VW-9000); and (**e**) snapshot of the interface position (*h* = 30.5 mm) and liquid plug recorded by using a smart phone (Videos S1 and S2 in the Supplementary Materials).

The pressure-sensing device is a commonly used glass capillary (outer diameter (o.d.) of 500 μm, inner diameter (i.d.) of 400 μm, and *L* = 95 mm in length) with one end sealed using epoxy resin adhesive. A T-shaped microchannel in a microfluidic chip is schematically shown in Figure 1a. The vertical channel is used as a side channel with a sealed inlet. The microchannel width and depth are *W* = 500 μm and *H* = 100 μm, respectively. The microfluidic chip (45 mm × 55 mm), which has never been used to study the mechanism of microdroplets generation, is made of PDMS and bonded with a glass plate on the bottom. There are four holes (diameter ~500 μm) on the T-shaped microchannel, where the pressure is to be measured (*P*_1_, *P*_2_, *P*_3_, and *P*_4_). *P*_2_ and *P*_3_ are the pressures in the side channel, which have the same value and are equal to the pressure (*p_conjection_*) at the conjection point of the T-shaped microchannel. The holes are drilled on the top PDMS slide (thickness *h*_w_ ~5 mm) using a hole puncher, and each hole is inserted with a sensing capillary vertically (Figure 1b). Both the inlet and outlet are connected to a 10-cm-length Poly Tetra Fluoro Ethylene (PTFE) tubing.

Deionized water (ρ = 1.0 g/cm^3^ and *μ* = 10^−3^ Pa·s) at 20 °C was infused via a syringe pump (Harvard Apparatus, PHD2000) at the flow rates (*Q*) ranging from 100 μL/min to 2667 μL/min. Scales are marked on the capillary to read the height (*h*) of the air-liquid interface directly (Figure 1c,d). The depth of the meniscus interface is below 100 μm, which is relatively very small. The height of the interface can be read with eyes vertically to the capillary (about 90°) for steady flow with a resolution of 0.5 mm. The movement of the interface can also be recorded by using a smart phone (Videos S1 and S2) with a better resolution of about 0.25 mm. The videos show that the meniscus can come back to the same position when the pressure is varied between two values. After 30 min and 100 times testing, the results can be repeated (deviation < 0.25 mm) without no drift and leakage. Then, the measured pressure can be read from a table calculating the *h*–pressure relationship utilizing the volumetric variation of trapped air in the capillary by the ideal gas law. We also employ a high-speed microscopic imaging system (Keyence, VW-9000) to record the air-liquid image during pressure increase. In order to make the measurement more accurate, the results are readout after about 5 min to make the flow system stable. Essentially, our trapped air compression method is similar to the methods of [54,55]. On the contrary, we replaced the fixed side channel and chamber with a reusable glass capillary as the sensor. Moreover, the effect factors have also been characterized in detail.

Our capillary-based pressure measurement method has the following advantages: (a) it does not need to fabricate additional microstructures, e.g., membranes, side channel, and chamber. The glass capillary is commonly used in labs and can be easily sealed with glue. (b) Just by inserting the capillary into a used PDMS microfluidic chip through a hole, pressure can be measured with the naked eye by directly reading the scales marked on it. Therefore, it is low-cost, electronic-free, and image-free and does not require auxiliary equipment for signal processing. (c) The *h*–pressure relationship is independent of the capillary diameter and the physical and chemical properties of the liquid. (d) The major advantage is that, once a bubble or liquid plug enters the capillary, it is easy to clear them, as the capillary can be pulled out from the microfluidic chip, which also means that it is robust and can be used for other microfluidic chips many times without recalibration.

## 3. Theory and Factors Analysis

For air confined inside a sealed capillary at constant temperature (*T*) and constant number of moles (*n*), its pressure (*p_g_*) can be calculated using the ideal gas law [58]:

(1) $$p_{g}V=nRT$$

where *V* is air volume and *R* is the gas constant (8.3145 J/mol·K).

**Figure 2:**
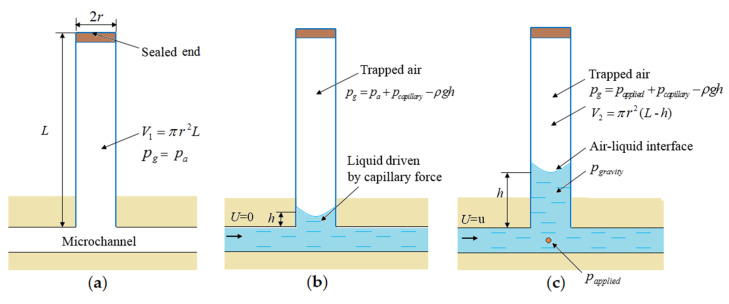
Schematic of the capillary-based pressure measurement using trapped air compression: (**a**) sealed capillary filled with air; (**b**) trapped air by a liquid plug driven by capillary force; and (**c**) balanced meniscus of the air-liquid interface under the applied pressure at the conjection point.

Initially, air at atmospheric pressure (*P_a_*) is contained in a one-end-sealed capillary and its volume is equal to that of the capillary (*V*_1_) (Figure 2a). When the microchannel is filled with liquid driven by capillary force, a little liquid plug is driven into the sealed capillary (Figure 2b). Therefore, the air inside the sealed capillary is trapped and compressed by the liquid plug driven by capillary force, inducing a capillary pressure drop *p_capillary_*. When an out pressure is applied to the liquid at the inlet, more liquid is forced into the capillary at the entrance of the capillary, which compresses the trapped air and induces increase of the air-liquid interface height (*h*) in the capillary. Finally, the air-liquid interface is balanced at an equilibrium position (Figure 2c). As no air escapes from the sealed glass capillary in the whole process, the air pressure inside the sealed capillary (*p_g_*) at constant *T* and *n* can be calculated using the ideal gas law as follows:

(2) $$p_{g}=p_{a}\times \frac{V_{1}}{V_{2}}=p_{a}\times \frac{L}{L-h}$$

In this expression, *V*_1_ = *πr*^2^*L* is the original volume of air and *V*_2_ = *πr*^2^(*L − h*) is the compressed volume of the air trapped inside the sealed capillary (Figure 2c), while the *r* and *L* are the capillary inner radius and length, respectively.

Once the air-liquid interface is at the equilibrium position (Figure 2c), the forces acting upon it must be balanced as in the following expression:

(3) $$p_{applied}-p_{gravity}=p_{g}-p_{capillary}$$

where *p_applied_* is the pressure in the microchannel at the entrance of the capillary and *p_gravity_* is the pressure caused by the liquid plug gravity in a vertical capillary. Finally, the applied liquid pressure at the entrance of the capillary can be calculated as the following formula:

(4) $$p_{applied}=p_{a}\times \frac{V_{1}}{V_{2}}+p_{gravity}-p_{capillary}$$

Figure 2c shows the schematic for pressure measurements with the effects of various factors. These factors affect the forces balance and measurement accuracy and must be considered. The impact ratios of these factors have been quantitatively evaluated in detail as follows.

### 3.1. Liquid Gravity

Driven by the pressure in the microchannel *p_applied_*, the air-liquid interface rises high in the vertical capillary (Figure 1c) and the pressure *p_gravity_* caused by the liquid plug gravity can be calculated using the following equation:

(5) $$p_{gravity}=\rho \;g\;h$$

where *ρ* is the liquid density and *g* is the acceleration of gravity. In the experiments, the value of *h* ranges from 0.5 to 41 mm. For water with *ρ* = 1000 kg/m^3^, *g* = 9.81 m/s^2^, and *h* = 41 mm, the maximum value of *p_gravity-max_* is

(6) $$p_{gravity}{}_{-\max}=\rho gh\;=\;402.2\;\mathrm{Pa}$$

Using Equation (2), the impact ratio of *p_gravity_* on *p_g_* can be calculated as

(7) $$S_{gravity}=\frac{p_{gravity}}{p_{g}}\times 100\%=\frac{\rho gh(L-h)}{p_{a}L}\times 100\%$$

when the air-liquid interface has the highest value (*h* =41 mm), the maximum value of *S_gravity-max_* is

(8) $$S_{gravity}{}_{-\max}=0.225\%$$

### 3.2. The Capillary Pressure Drop

Due to the interfacial tension and inner capillary radius varying from *r* = 200 μm, the capillary pressure drop *p_capillary_* occurring on the air-liquid interface, will result in an error in the pressure measurement (Figure 1b). Therefore, it is necessary to consider the influence of *p_capillary_*, which can be calculated by the Young–Laplace equation [59,60]:

(9) $$p_{capillary}=\frac{2\sigma \cos\theta }{r}$$

where *σ* is the liquid surface tension, *r* is the capillary inner radius, and *θ* is the contact angle of the liquid on glass, which may advance (*θ* = *θ_a_*) or recede (*θ* = *θ_r_*) depending on whether the applied pressure increases or decreases. With *σ* = 7.28 × 10^−2^ N/m and *θ* = 60° at 20 °C for water in the experiments (Figure 1d), the maximum value of *p_capillary_* with *r* = 200 μm is

(10) $$p_{capillary}=\frac{2\sigma \cos\theta }{r}=364Pa$$

In Figure 1b, although the applied pressure *p_applied_* is *p_a_*, a small amount of liquid wicks into the sealed capillary induced by the capillary force. Substituting Equations (2), (5), and (10) into Equation (10), the balanced equilibrium position of the air-liquid interface (*h_c_*) in the sealed capillary can be calculated by the following equation:

(11) $$p_{capillary}=p_{a}\times \frac{h_{c}}{L-h_{c}}+\rho gh_{c}$$

Then, we can obtain the maximum value *h_c_* of 0.25 mm, which will decrease as the air-liquid interface height increases. According to Equation (2), in the experiments, the minimum measured value of *p_g_* is 101.6 kPa (*h* = *h*_c_ = 0.25 mm). As the *p_capillary_* value is fixed and does not change with the air-liquid interface during the measurements, the impact ratio of *p_capillary_* on *p_g_* has the maximum value of

(12) $$S_{capillary}=\frac{p_{capillary}}{p_{g-\min}}=\frac{364\times L\mathrm{Pa}}{p_{a}(L-h_{c})}=0.36\%$$

### 3.3. Air Dissolution in Water

In order to improve the measurement accuracy, the change of air solubility induced by pressure increasing from *p*_a_ to *p*_g_ should be considered, which can be calculated using Henry’s Law [61]:

(13) $$C=K_{H}p_{g}$$

where *C* is the solubility of air at a fixed temperature in a particular solvent and *K* is Henry’s law constant, which decreases with increased temperature, as shown in Table 1.

At temperature of 20 °C, the volume of air dissolved in the liquid plug is thus given as follows:

(14) $$V_{air-drop}=K_{H}(p_{g}-p_{a})V_{plug}=\frac{h}{L-h}K_{H}\pi r^{2}hp_{a}$$

Therefore, the actual gas pressure should be a little lower than the calculated value of *p_g_* using the ideal gas law. The maximum impact ratio of the pressure drop *p_air-drop_* caused by the dissolution of gas with *h* = 41 mm is

(15) $$S_{air-drop}=\frac{p_{air-drop}}{p_{g}}=\frac{V_{air-drop}}{V_{gas}}=\frac{K_{H}p_{a}\pi r^{2}h^{2}}{\pi r^{2}(L-h)L}=0.553\%\;$$

### 3.4. Temperature Changes

Temperature also has influences on the liquid surface tension (Table 2), the fluid viscosity and density, the air solubility, and the ideal gas law, which lead to measurement errors. With temperature changing from 10 °C to 30 °C, the impact rate of *σ* is

(16) $$S_{\sigma }=\frac{\sigma_{10{}^{\circ}C}-\sigma_{30{}^{\circ}C}}{\sigma_{10{}^{\circ}C}}\times 100\%=4.04\%$$

Considering the impact ratio *S_capillary_* of 0.36%, the error caused by variation of *σ* can be ignored. Furthermore, as the fluid in the capillary is static, the viscosity variation effect can also be ignored.

According to the ideal gas law (Equation (1)), temperature changes will induce pressure variations in the trapped air. Assuming that the sealed air volume is constant, with a temperature variation Δ*T* of ±10 °C from 20 °C, the temperature-induced pressure variation ratio can be calculated as

(17) $$S_{\Delta T}=\frac{p_{g}\Delta T}{p_{g}T_{20℃}}=\frac{\Delta T}{T_{20℃}}=\frac{\pm 10}{273.15+20}\times 100\%=\mp 3.41\%$$

The results show that the temperature variation Δ*T* has an effect on pressure measurement, leading to an error of 3.41% in the actual applied pressure *p_applied_*. Please note that the temperature variation also has effects on air solubility *C*, expressed in Equation (13) and shown in Table 1. Considering Equation (15), the calculated temperature-induced impact ratio of gas dissolution on pressure is less than 0.2%. 

The finally obtained total impact ratios of these factors on the measured pressure *p_applied_* are

(18) $$S_{total}=-S_{gravity}+S_{capillary}+S_{air-drop}+S_{\Delta T}$$

Assuming that temperature is constant, according to the above equation, *S_total_* is less than 0.52%, which is relatively very small. Therefore, Equation (4) can be simplified as Equation (2). The calculated result is consistent with the conclusions of Srivastava and Burns [54]. The influence factors have been quantitatively characterized in detail in our study. Please note that the theoretical calculated pressure results can be independent of the capillary inner diameter, which means that capillaries with other inner diameters can also be used (200–800 μm), which covers the characteristic width scale of most microfluidic systems. Please note that the capillary diameter is much larger than the side indicator channel of Ref. [53], where the side channels range from 10 to 100 μm in wide with a deep of 20 μm and the capillary forces on the meniscus become significant. When the temperature changes to actual temperature of *T*_a_, the modified equation for *p_applied-T_* is:

(19) $$p_{applied-T}=p_{applied-20{}^{\circ}C}\times \frac{T_{a}+273.15}{20+273.15}$$

Therefore, the above calculated total systematic error is relatively small and acceptable in most microfluidic systems. In addition, although the above formula is inconsistent with the theoretical results, the measurement of pressure drop of different points still has a high resolution due to mutual cancellation.

### 3.5. Theory of Pressure Drop

According to the Hagen–Poiseuille law [26], pressure drop Δ*p* between two locations inside a rigid rectangular channel at low Reynolds number can be approximately calculated as

(20) $$\Delta p=\frac{aQ\mu L}{WH^{3}}$$

(21) $$a=12{\left[1-\frac{192H}{\pi^{5}W}\tanh(\frac{\pi W}{2H})\right]}^{-1}$$

where *Q* is the mean volumetric flow rate, *H* and *W* are the depth and width of the channel, *L* is the distance between the two points across which the pressure drop occurs, *μ* is the liquid viscosity, and *a* is a constant specific to channel geometry and can be calculated by Equation (21). The channel dimensions *H, W, Q, μ*, and *L* are known, so Δ*p* can be calculated.

In fact, for elastic microchannels, flow–elasticity coupling makes the flow-pressure distribution in the channel nonlinear. According to the flow rate–pressure drop relation in deformable shallow microfluidic channels [17], pressure drop Δ*p* between two locations inside an elastic rectangular channel at low Reynolds number can be approximately calculated as

(22) $$Q\approx \frac{H^{3}W\Delta p}{a\mu L}[1+\frac{3}{160}{\left(\frac{W}{h_{w}}\right)}^{3}(\frac{W}{H})(\frac{\Delta p}{E})]$$

where *h_w_* is the top wall’s thickness and *E* is Young’s modulus of the material. According to this formula, the relationship between flow rate and pressure drop is nonlinear in a unilateral deformable channel, which has become one of the hot topics concerned by many researchers [17,26].

## 4. Simulation Method

**Figure 3:**
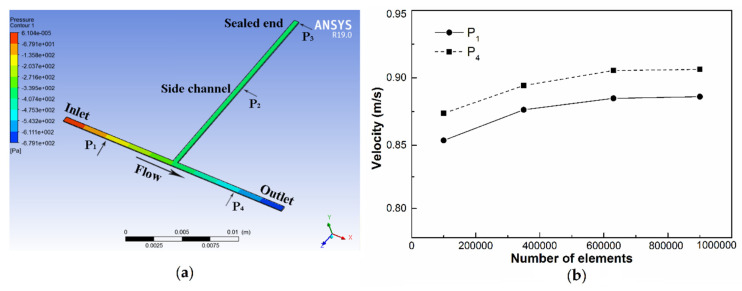
(**a**) Pressure contour plot for the main structure of the one-end-sealed capillary pressure sensor and (**b**) effect of the number of grids on the velocity, evaluated at the center points of two different positions of the main channel (*Q* = 2000 μL/min).

The main structure of the one-end-sealed capillary pressure sensor is simulated by ANSYS (R19.0) in order to verify the structural principle. We first create and modify the geometry in SolidWorks (SolidWorks Corp. 2018) for analysis. Meshing is the second essential part of the simulation process. Through optimized meshing, accurate results of flow rates and speed are obtained. Setup, solution, and results optimization in ANSYS are the final parts of the simulation process. The modal simulation results of the contours of pressure are shown in Figure 3a. Please note that, as fluid–solid interaction (FSI) simulation is complex, to simplify the simulation process, slight deformation of the elastic PDMS channel at high pressures has not been simulated.

### 4.1. Geometry

The geometries of the microfluidic T-junctions in the present work are shown in Figure 1a. The widths of the main and side channels are both 500 μm, and the measuring point is 5 mm from the inlet and outlet of the main channel. A fully developed laminar flow can be obtained after a distance of [62]

(23) $$L_{e}=D_{h}(\frac{0.6}{1+0.035Re}+0.056Re)$$

*Re* and *D_h_* are the flow Reynolds number and hydraulic diameter of the microchannel and can be expressed as follows, respectively,

(24) $$Re=\frac{\rho UD_{h}}{\mu }$$

(25) $$D_{h}=\frac{2WH}{W+H}$$

where *U* is the mean velocity in the microchannel and *D_h_* is 167 μm. The calculated *Re* ranges from 9.3 to 148. In this study, the maximum entrance length needed for a fully developed flow corresponding to *Re* of 148 is about 1.4 mm. Therefore, the lengths of the upstream and downstream are fixed at 5 mm to ensure the flow is fully developed in the microchannel.

### 4.2. Governing Equations

In the process of numerical simulation, the fluid is considered incompressible, with a fixed viscosity, and the influence of gravity is ignored. The governing equations are the continuity Equation (26) and incompressible Navier–Stokes (N-S) Equation (27). A finite volume method was utilized to turn the governing partial differential equations into a system of algebraic equations, which are numerically integrated over each of the computational cells using a collocated cell-centered variable arrangement.

(26) $$\frac{\partial \rho }{\partial t}+\nabla \cdot (\rho \vec{V})=0$$

(27) $$\rho \frac{D\vec{V}}{Dt}=\rho \vec{f}-\nabla p+\mu \nabla^{2}\vec{V}$$

where *t*, $\vec{V}$, and $\vec{f}$ are the time, velocity, and gravitational force, respectively.

For a solution algorithm, the pressure implicit with splitting of the operator scheme is used for pressure–velocity coupling, the pressure staggering option scheme is adopted for pressure interpolation, and the second-order upwind difference method is adopted for the momentum equation. As the boundary conditions, uniform inlet velocities are set to the inlets of main channels and fixed atmospheric pressure is set to the outlet. No slip boundary condition was applied at the walls, and a constant contact angle is used as the wall wetting condition. The time step is carefully selected to ensure that the Courant number is lower than 0.25. To find an approximate solution, a residual value less than 10^−6^ was used for continuity and momentum equations.

### 4.3. Grid Independency Study and Validation

**Figure 4:**
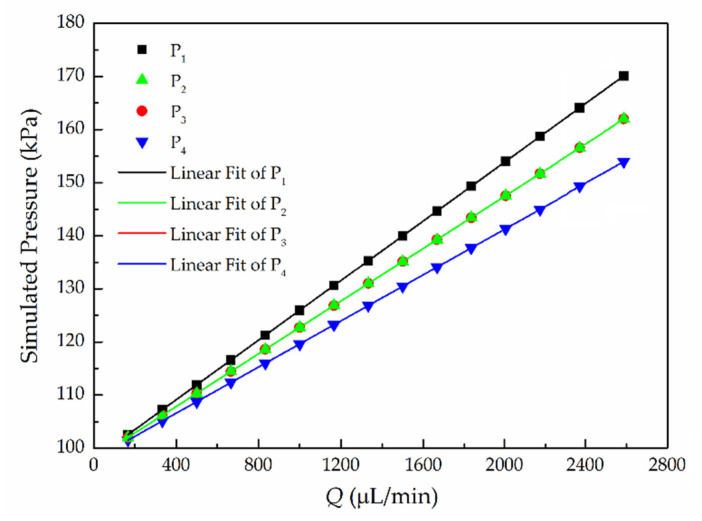
Simulated results of the relationship between flow rate *Q* and the pressure *p_g_* of points 1–4.

The simulation domain was meshed using hexahedral cells. To show independency of results from the number of computational elements, we carried out numerical simulations on a representative microchannel to evaluate the sensitivity of the number of grids on the velocity. We performed simulations for a fixed flow rate (*Q* = 2000 μL/min) and two different positions of the main channel (inlet and outlet of the microchannel). The results are shown in Figure 4. We find that, for the number of grids <350,000, variation in the velocity is smaller than 3%, while for the number of grids >600,000, it is smaller than 0.2%. Therefore, it was finally determined that the number of grids is 630,000, the grid cell size was 0.01 mm, and the grid quality reached 0.98.

## 5. Results and Discussion

### 5.1. Simulation Analysis

The simulation results show that pressure increases at the input and reduces at the output of the measurement channel, while it remains constant in the side channel (Figure 3a). The pressure at a certain point can be obtained directly, and two points of pressure can be calculated as the pressure drop (Δ*p*). Moreover, the relationship between the pressure drop and the distance is obtained.

Figure 4 presents a standard linear relationship between pressure *p*_g_ and flow rate at each point, which increases linearly with *Q* increasing (100 μL/min to 2667 μL/min). The simulated pressure values of points 2 and 3 are the same at the given flow rates, indicating that the static hydraulic pressure in the side channel remains constant. Moreover, it can be seen that the pressure drop at two equidistant points is almost the same.

### 5.2. One-Point Pressure Measurement

**Figure 5:**
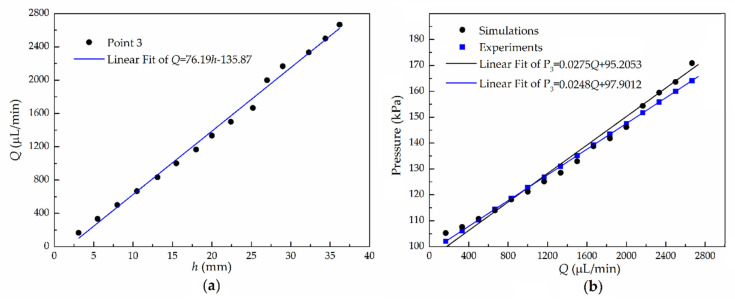
(**a**) Experimental results of the relationship between the air-liquid interface height (*h*) and flow rates (*Q*) and (**b**) comparison of experimental results and simulated results for *Q*–*p_g_* fitting line.

As the PDMS microfluidic chip has never been used for droplets generation previously, hole 3 was never used as an inlet of dispersed phase liquid. Therefore, to verify the measurement method, pressure at hole 3 was first measured by inserting a one-end-sealed capillary before holes 1, 2, and 4 were drilled. Before inserting the capillary, the side channel was filled with water, making sure that there were no air bubbles. Then, a one-end sealed capillary was inserted into hole 3 and glued around. Fixed flow rates *Q* were applied to a pipe connected to the inlet of the microchannel. The relationship between the flow rates *Q* and the height of the air-liquid interface (*h*) was recorded in experiments, as shown in Figure 5a. Please note that, at *Q* < 3000 μL/min, according to Equation (22), as deformation of the channel is relatively small, the relationship between the flow rate and pressure is still linear for this microfluidic chip. The relationship between *Q* and the corresponding air pressure *p_g_* is shown in Figure 5b. It can be found that the relationships of *h–Q* and *Q*–*p_g_* are both nearly linear. Then, we compared the experimental results with the simulated results, which were also in good agreement, conforming to the pressure versus flow rate relationship. 

Moreover, at relatively high flow rates (>2000 μL/min), the actual fluid pressure of the experiment results is a little lower than that of the simulation results due to the slight elastic deformation of the PDMS channel. Therefore, by observing the air-liquid interface height, it is easy to obtain the actual liquid pressure and flow rates in the experiments. Using a smart phone, the movement of the interface position can be recorded clearly. In Video S1, the *h* varies from 25 mm to 41 mm, while in Video S2, *h* varies from 18 mm to 30 mm. When the pressure change significantly, the *h* will reach a steady position within 2–3 seconds, which is dependent on the flow systems (the pump, tubing, microchannel, etc.). 

The results in Figure 5 prove that the capillary-based pressure-sensing method is feasible and simple. Without additional equipment and results postprocess, just by reading the height of the air-liquid interface in the capillary, the fluid pressure can be directly measured. The measured pressure range in point 3 is from 101.6 kPa to 167 kPa, while the flow rates range from 100 μL/min to 2667 μL/min. These pressure ranges and flow rates are commonly employed in microfluidic systems. Higher flow rates and pressures may cause significant deformation of the microchannel or detachment the PDMS layer from the glass slide. Moreover, the capillary sensor is convenient and reusable for short-term (hours) pressure measurement in other applications, where the abilities of quick response (>1 Hz) and high sensitivity (<0.5 kPa) are not highly demanded. Furthermore, to realize the continuous pressure measurement capability of this method for some biochemical applications, one can employ a smart phone or high-speed microscopic imaging system and image postprocessing software. In addition, to measure very small pressure variation induced by, e.g., passing droplets or cells, the capillary diameter should be as small as that in Ref [53] and the effect factors should be characterized again.

### 5.3. Multiple-Points Pressure Measurement

Liquid pressure at multiple points of the microchannel can be measured by using more sensing capillaries, and the pressure drop can also be obtained. Four capillaries were inserted into each of holes 1–4 drilled along the T-shaped microchannel. The microchannel length and positions of the holes are shown in Figure 1b. Capillaries 1 and 4 align along the flow direction to measure the pressure drop in the main microchannel, while capillaries 2 and 3 are used to measure the static pressure in the middle point of the microchannel. The measured air-liquid interface heights of these four capillaries with different flow rates are shown in Table 3.

**Figure 6:**
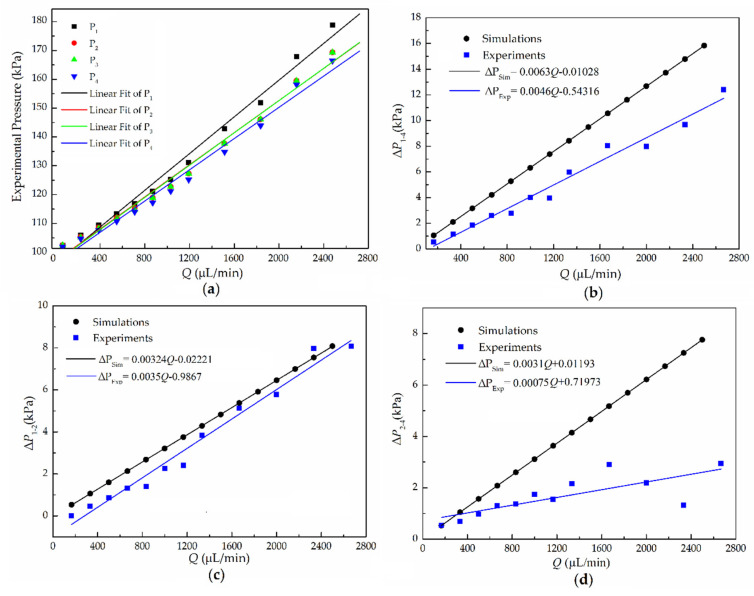
(**a**) Experimental results of the relationship between flow rate *Q* and pressure *p_g_* and (**b**–**d**) comparison of experimental results (blue) and simulated results (black) for pressure drop between (**b**) capillaries 1 and 4, (**c**) capillaries 1 and 2, and (**d**) capillaries 2 and 4 versus the flow rate.

The results in Figure 6a show that the relationship between flow rate *Q* and pressures in the four capillaries fits a straight line and the pressure range in point 1 is from 101.6 kPa to 178 kPa. It is also found that the measured pressure in capillaries 2 and 3 are almost the same for the same flow rate. In Figure 6b,c, the pressure drops (Δ*p*_1–4_ and Δ*p*_1–2_) and flow rates also have a linear relationship. Pressure drop (Δ*p*_1–4_) as low as 0.25 kPa was obtained in an operating range from 0.5 kPa to 12 kPa. Consistent with the simulation results, the experimental results of the pressure drop increase linearly as the flow rate increases. The results show a relatively good agreement between the results of experimental and ANSYS simulations. 

In Figure 6b, for deformation of the PDMS channel, the pressure drop of the experimental results are lower than that of the simulation results. At *Q* = 2000 μL/min, the Δ*p*_1-4_ values are 12.8 kPa and 8.1 kPa, respectively, which means that elastic deformation of the channel cannot be ignored if precise pressure and flow velocity are significant to the microfluidic system. In Figure 6d, as the pressure drop is relatively small, the measured Δ*p*_2-4_ is not strictly linear with the flow rate. Moreover, the slopes of the fitting lines of both the simulation and experimental results decreases in Figure 6b–d. The results prove that the multiple points of capillary sensing can measure not only pressure on various points but also pressure drops.

### 5.4. Improvement and Application 

In order to make the simple capillary-based method more convenient, we calculate and calibrate the relationship between the air-liquid height and the measured pressure using the Equations (4) and (18). We mark scales on the capillary schematically shown in Figure 2c. The conversion formulas of the liquid heights (*h*), the actual pressure (*p*_applied_), and relative pressure (*p*_relative_) can be expressed as

(28) $$p_{applied}=p_{a}\frac{L}{L-h}\times (1-0.52\%)=\frac{100.79\times 95}{95-h}k\mathrm{Pa}$$

(29) $$p_{relative}=p_{a}\frac{h}{L-h}\times (1-0.52\%)=\frac{100.79\times h}{95-h}k\mathrm{Pa}$$

According to Table 4, the pressure value can be measured directly by reading the height of the air-liquid interface (*h*). Therefore, this method is very convenient and simple compared with other methods which need extra optical and electronic equipment. In these experiments, the highest value of *h* is 40 mm according to a pressure range of 101.6–174 kPa. If the capillary was used for microfluidic chips made of glass, a wider pressure range can be achieved. Moreover, the position of the air-liquid interface can be clearly read with a resolution of 0.5 mm using eyes. The pressure measurement resolution of this one-end-sealed capillary can be calculated as

(30) $$e=p_{a}L(\frac{1}{L-h-0.5}-\frac{1}{L-h})\times 99.48\%=\frac{100.79L}{2(L-h-0.5)\times (L-h)}k\mathrm{Pa}$$

According to this formula, the pressure resolution varies from 0.554 kPa to 1.657 kPa with the height of the air-liquid interface increasing from 1 mm to 41 mm. If we use a smart phone to record the interface position, the pressure resolution will be improved.

**Figure 7:**
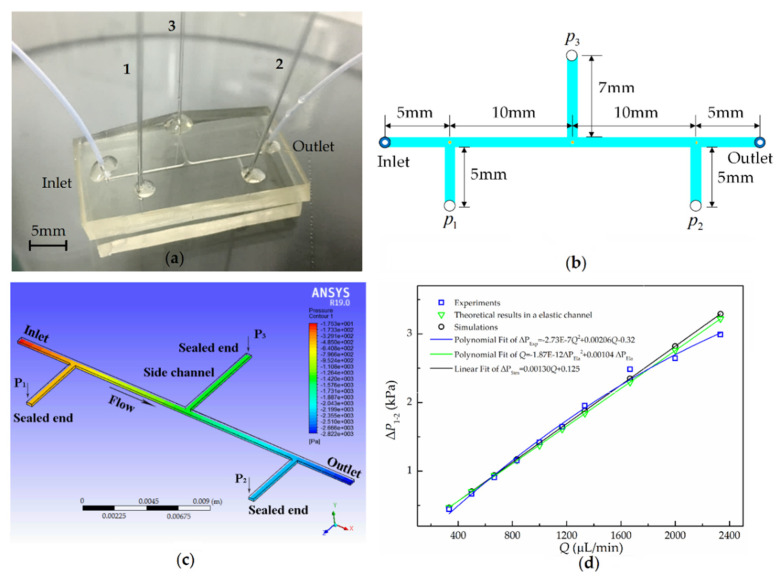
(**a**) Snapshot of the polydimethylsiloxane (PDMS) pressure-drop microfluidic chip with three sensing capillaries; (**b**) schematic diagram of the microchannel with three holes, where three vertical channels are used as sealed side channels; (**c**) pressure contour plot for the PDMS pressure-drop microfluidic chip with three sensing capillaries; and (**d**) comparison of the experimental results (blue square), simulated results (black circle), and theory results in elastic channels (green triangle) for pressure drop between capillaries 1 and 2 versus the flow rate.

To prove the universality of our method, we further measured and obtained three-point pressure and pressure drop in another microchannel. The T-shaped microchannel in the microfluidic chip is shown in Figure 7a. Three vertical channels were used as side channels with the inlet holes (diameter ~500 μm) inserted by a sensing capillary. The microchannel width and depth were *W* = 480 μm and *H* = 200 μm, respectively. Both the bottom and the top slides of the microfluidic chip were made of elastic PDMS, and the thickness was *h*_w_ = 3 mm. Figure 7b schematically shows the microchannel structure and the lengths of the three side channels with inlet holes, where the pressure is to be measured (*P*_1_, *P*_2_, and *P*_3_). The simulation results of the contours of the pressure are shown in Figure 7c. Similarly, pressure near the inlet is high and decreases along the microchannel, while pressure in the side channel remains constant. In addition, the relationship of the pressure drops and the distance between the three points is obtained.

To make a quantitative comparison between the results of simulation, theory, and experiments, Figure 7d shows the plots of the flow rate–pressure drop (*Q*-Δ*p*) relation. The theoretical results are based on the flow–elasticity coupling asymptotic theory [17], while the experimental results are obtained using our one-end-sealed capillary method. The simulation results show that the pressure drop increases linearly with the flow rate, which is consistent with the theory based on the Hagen–Poiseuille law [26] mentioned above, while the experimental and elastic theoretical fitting results are curved and nonlinear. Through comparative analysis, we find that, when *Q* < 1600 μL/min, the results of simulation, experiment, and elastic theory basically coincide, indicating that deformation can be ignored. Affected by elastic materials, when *Q* > 2000 μL/min, the elastic theory and experimental results begin to decrease.

When *Q* = 2400 μL/min, the experimental result is obviously lower than the results of the elastic theory, which is used for microchannels with a unilateral PDMS slide [17]. We speculate that this is caused by elasticity of the PDMS slides on the both sides of our microfluidic chip. The results show that microchannels with elastic material slides on both the top and bottom sides will cause more expansion of the cross-sectional area and that the pressure drop within it will be smaller than that calculated by the unilateral elastic theory. On the other hand, for deformation of the top and bottom PMDS walls, a smaller pressure gradient is needed to achieve the same volumetric flow rate. It can be seen that our method can reasonably analyze the nonlinearity of the flow-pressure drop relationship caused by channel deformation.

## 6. Conclusions

We have presented a simple capillary-based method to measure liquid pressure inside a microchannel that applies to multiple conditions and is easy to operate and read. Various effect factors (liquid gravity, capillary force, air solubility, and temperature) have been quantitatively investigated in detail, verifying accuracy of this method. Pressure measurements have been obtained through experiments, and the errors have been analyzed. Simultaneously, we constructed a finite volume model of the microchannel used in our experiments and simulated the corresponding pressure distribution. A complete evaluation was performed by comparing the experimental, theoretical, and simulation results. The results prove that the capillary-based method can measure not only pressures at multiple points but also the pressure drops. Using this method, we have measured the nonlinearity of the flow-pressure drop relationship caused by channel deformation. In summary, the capillary-based pressure sensor is simple, unpowered, easily fabricated and can be a generally used method, which has similarities to the convenient clinical thermometer and potentials for various microfluidic applications. 

## Figures and Tables

**Table 1:** Solubility coefficient of air in water at different temperatures.

T (°C)	0	10	20	30	40	50
K_H_ (L/kPa·m^3^)	0.285	0.218	0.180	0.158	0.135	0.120

**Table 2:** Water surface tension at different temperatures.

T (°C)	0	5	10	15	20	25	30
σ (×10^−2^ N/m)	7.56	7.49	7.42	7.35	7.28	7.21	7.12
ρ (kg/m^3^)	999.9	1000	999.7	999.1	998.2	997.1	995.7
μ (×10^−3^ Pa·s)	1.787	1.519	1.307	1.140	1.002	0.890	0.798

**Table 3:** The corresponding relationships between flow rates and air-liquid interface heights in 4 capillaries.

Q (μL/min)	h_1_ (mm)	h_2_ (mm)	h_3_ (mm)	h_4_ (mm)
167	1	1	1	0.8
333	4	3.6	3.4	3
500	7	6.3	6	5.5
667	10	9	8.8	8
833	12.5	11.5	11	10.5
1000	15.5	14	14	12.8
1167	18	16.5	16.5	15.5
1333	21.5	19.3	19.3	18
1667	27.5	25	25	23.5
2000	31.5	29	29	28
2333	37.5	34.5	34.5	34
2667	41	38	38	37

**Table 4:** Relationship between the height of air-liquid interface and pressures.

h (mm)	0	1	2	3	4	5	6	7	8	9	10	20	30	40
*p_applied_* (kPa)	101	102	103	104	105	106	107	108	110	111	112	127	147	174
*p*_relative_ (kPa)	0	1.07	2.16	3.27	4.41	5.58	6.77	7.99	9.23	10.51	11.8	26.8	46.4	74.0

## Supplementary Materials

The following are available online at https://www.mdpi.com/2072-666X/11/10/914/s1, Video S1: h from 18 to 30 mm with scales using smart phone, Video S2: h from 25 to 41 mm with a rule using smart phone.
